# Regulatory T-cells from pancreatic lymphnodes of patients with type-1 diabetes express increased levels of microRNA miR-125a-5p that limits CCR2 expression

**DOI:** 10.1038/s41598-017-07172-1

**Published:** 2017-07-31

**Authors:** Guido Sebastiani, Giuliana Ventriglia, Angela Stabilini, Carlo Socci, Cristina Morsiani, Andrea Laurenzi, Laura Nigi, Caterina Formichi, Bechara Mfarrej, Alessandra Petrelli, Georgia Fousteri, Todd M. Brusko, Francesco Dotta, Manuela Battaglia

**Affiliations:** 1 Diabetes Unit, Department of Medicine, Surgery and Neuroscience, University of Siena, Siena, Italy; 2 Fondazione Umberto Di Mario ONLUS c/o Toscana Life Science, Siena, Italy; 3 Diabetes Research Institute, IRCCS San Raffaele Scientific Institute, Milan, Italy; 4 Department of Surgery, IRCCS San Raffaele Hospital, Milan, Italy; 5 Department of Internal Medicine, IRCCS San Raffaele Hospital, Milan, Italy; 6 Department of Pathology and Laboratory Medicine, Diabetes Institute, Gainesville, FL USA; 7 Present Address: Interdepartmental Centre “L. Galvani” for Integrated Studies of Bioinformatics, Biophysics and Biocomplexity (CIG), University of Bologna, Bologna, Italy

## Abstract

Autoimmune type 1 diabetes (T1D) is thought to be caused by a defective immune regulation with regulatory T (Treg) cells playing a fundamental role in this process. Tolerance mechanisms depend on tunable responses that are sensitive to minor perturbations in the expression of molecules that can be carried out by multiple epigenetic mechanisms, including regulation by microRNAs. In this study, microRNA expression profile was investigated in Treg cells isolated from peripheral blood (PB) and from pancreatic draining lymph nodes (PLN) of T1D patients and non-diabetic subjects. Among 72 microRNAs analyzed, miR-125a-5p resulted specifically hyper-expressed in Treg cells purified from PLN of T1D patients. TNFR2 and CCR2 were identified as miR-125a-5p target genes. Elevated miR-125a-5p was detected in Treg cells isolated from PLN but not from PB of donors with T1D and was associated with reduced CCR2 expression. A specific beta-cell expression of the CCR2-ligand (CCL2) was observed in the pancreata of cadaveric donors, suggesting that beta-cells are prone to attract CCR2^+^ Treg cells. These novel data propose a mechanism, occurring in PLNs of T1D patients, involving increased expression of miR-125a-5p on Treg cells which results into reduced expression of CCR2, thus limiting their migration and eventual function in the pancreas.

## Introduction

MicroRNAs are a class of small endogenous non-coding RNAs (19–24 nucleotides long) that regulate gene expression post-transcriptionally by mRNA translational repression or decay. They specifically bind to the 3′UTR of mRNA target in a sequence specific manner, in respect of mRNA secondary structure itself^1, 2^. In the light of their function as gene expression regulators, microRNAs have been widely linked to several biological processes (e.g. cell cycle, apoptosis, differentiation and development) and consequently reported to drive or to be associated to alterations in several diseases. Moreover, microRNAs have been reported to act as regulators of immune homeostasis^3, 4^ showing specific expression patterns among different cell types of the immune system, including T regulatory (Treg) cells^5, 6^.

Type 1 diabetes (T1D) is a chronic autoimmune disease, involving impaired immune-regulation, characterized by the specific destruction of pancreatic beta-cells leading to altered glucose homeostasis^7^. In healthy individuals, autoreactive T cells are controlled by peripheral tolerance mechanisms, among which Treg cells have emerged as key mediators^8^. Understanding the cell-intrinsic cues that permit regulation in lymphocytes, and therefore control of autoimmunity, requires an understanding of the transcriptional and post-transcriptional regulation of gene expression in these cells. As previously demonstrated, the deregulation of Treg cells suppressive function more than their peripheral blood frequency, may be a factor influencing the pathogenesis of human T1D^9^. Additionally, we have previously demonstrated that pancreatic draining lymph nodes (PLN) from patients with T1D retain Treg cells (CD4^+^CD25^bright^) epigenetically imprinted to have a Treg phenotype but that, for still unknown reasons, are functionally defective *in vitro* ^10^.

Aim of this work was to explore the possibility that post-transcriptional regulatory mechanisms influence Treg cells when residing in the PLN but not when circulating in the periphery of patients with T1D. To this purpose, microRNA expression profile was investigated in Treg and in T conventional (Tconv) cells purified from peripheral blood (PB) and from pancreatic lymph nodes (PLN) of patients with T1D.

## Results

### MicroRNAs expression profile analysis validation from 200 sorted Treg cells

In order to analyze microRNA expression profiles in Treg cells sorted from peripheral blood and pancreatic lymph nodes (PLN) of patients with T1D and of non-diabetic subjects (Supplementary Figure 1A), we adopted a method that allows the quantification of microRNAs in as low as 200 cells^11, 12^. Although this method was shown to be sensitive and specific, it may lead to some degree of variability in the number of detected microRNAs. In order to avoid potential artifacts, only those microRNAs that were identified in all replicates of all samples were included in our analysis (i.e., 72 microRNAs out of 384 tested). In addition, to verify the reliability of this method, microRNA expression in 200 and 5000 Treg cells purified from peripheral blood of 3 non-diabetic donors were compared. An optimal degree of correlation between microRNA expression in 200 and 5000 cells was observed (Supplementary Figure 1B), thus confirming that 200 Treg cells are sufficient for accurate microRNA profiling. Moreover, to further validate the microRNAs analysis method, we tested the expression of two microRNAs (miR-31 and miR-125a-5p) previously found enriched in Tconv cells respect to Treg cells; the results confirmed the higher expression of these microRNAs in PB Tconv cells respect to PB Treg cells thus validating the method efficiency in detecting microRNAs expression differences in 200 sorted cells (Supplementary Figure 2).

### MicroRNA miR-125a-5p is upregulated in Treg cells sorted from T1D PLN

**Figure 1:**
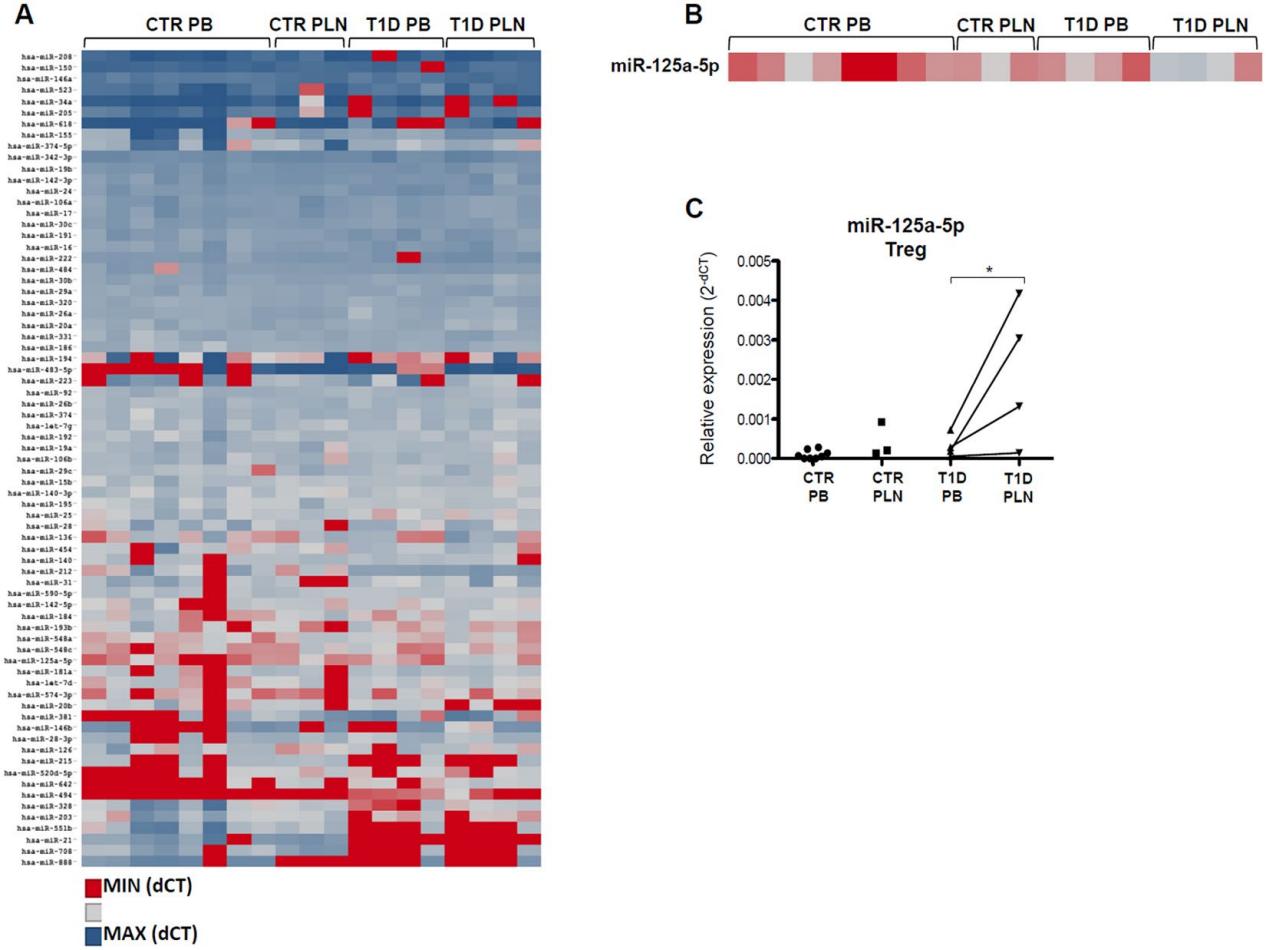
MicroRNA expression profiles reveal that miR-125a-5p is over-expressed in Treg cells isolated from PLN of patients with T1D. (**A**) microRNA expressions in Treg cells isolated from peripheral blood (PB) of non-diabetic control (CTR) donors (n = 8), pancreatic lymph nodes (PLN) of controls (n = 3) and from PB and PLN of patients with T1D (n = 4) is reported in the hierarchical heatmap. Expression values are reported as delta cycle threshold (dCT) values normalized using three different small RNAs (RNU6, RNU44, RNU48) and represented by scale colour of normalized dCT values [Red- low expressed microRNAs; blue- highly expressed microRNA]. Euclidean complete linkage clustering method was used to infer the data in the heatmap clustering graph. (**B**) Zoom-in of heatmap graph reporting miR-125a-5p expression values [Red- low expressed microRNAs; blue- highly expressed microRNA]. (**C**) The expression levels of miR-125a-5p were extrapolated from the heat map shown in panel B. Dots connected by a line represent paired samples. *p < 0.05 paired 2-tailed “Student t-test” on dCt.

MicroRNAs were measured in 200 Treg cells isolated from PB and PLN of control non-diabetic subjects and from patients with T1D (Supplementary Table 1) (Fig. 1A). Treg cells purified from PLN of patients with T1D were impaired in their *in vitro* suppressive capacity, while Treg cells isolated from blood of the same patients were functional as well as those isolated from both PLN and PB of control non diabetic subjects (Supplementary Figure 3), confirming our previous findings^10^. In an attempt to identify the microRNA(s) potentially responsible for this T1D PLN-specific Treg-cell dysfunction *in vitro*, we selected those microRNAs that were differentially expressed only between Treg cells derived from T1D PLN respect to T1D PB but not between PB and PLN of non-diabetic control subjects. MiR-125a-5p was the only microRNA, among the 72 analyzed, that was significantly and specifically upregulated in Treg cells residing in PLN vs Treg cells in PB of T1D patients, as well as vs Treg cells isolated from PLN and from PB of control non-diabetic donors (Fig. 1B and C).

**Figure 2:**
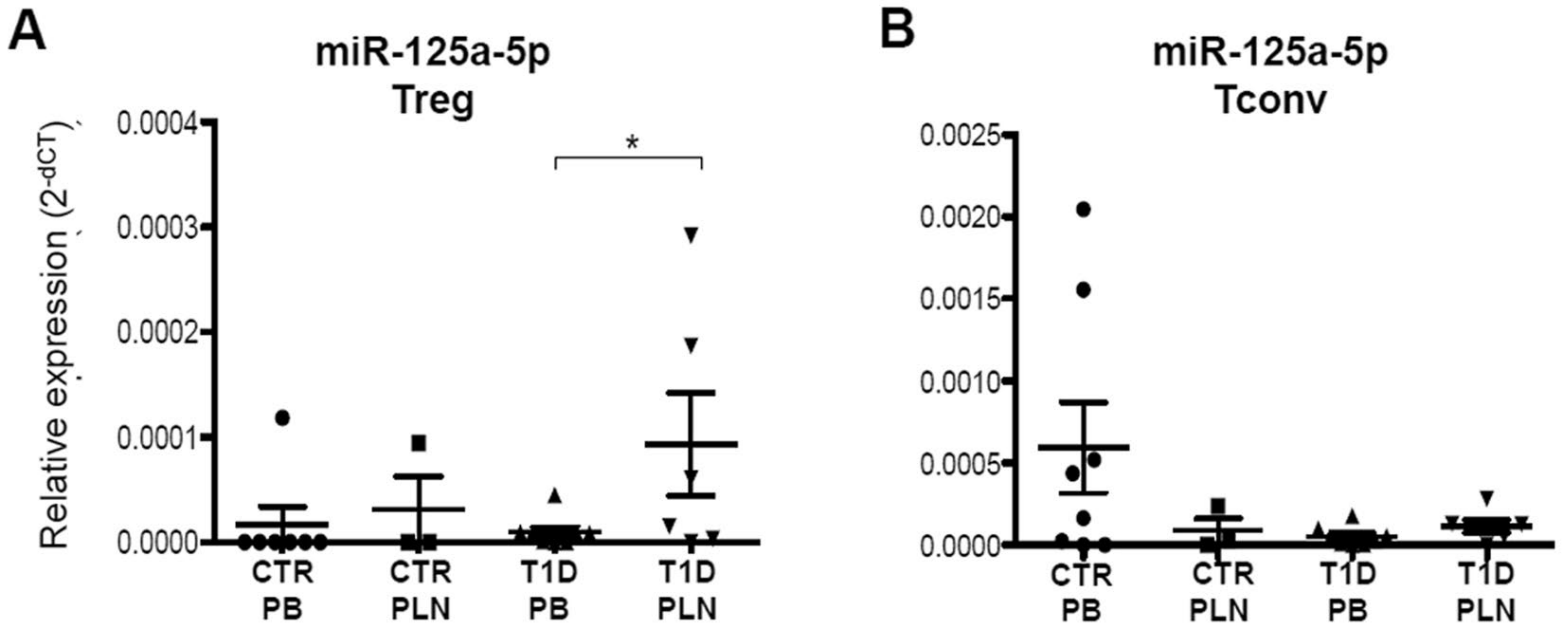
miR-125a-5p is selectively hyper-expressed in Treg cells isolated from PLN of patients with T1D. miR-125a-5p expression was measured by real time-PCR in Treg cells **(A)** and Tconv cells **(B**) purified from peripheral blood (PB) of non-diabetic control (CTR) donors (n = 8), pancreatic lymph nodes (PLN) of controls (n = 3) and from PB (n = 8) and PLN (n = 5) of patients with T1D. Expression values are reported as 2^-delta cycle threshold (2^−dCT^) values normalized using three different small RNAs (RNU6, RNU44, RNU48). *p < 0.05 student’s *t test*.

To confirm this finding, miR-125a-5p expression was measured by real time-PCR in Treg cells isolated from PB and PLN of an additional cohort of control non-diabetic donors and of patients with T1D (donor characteristics in Supplementary Table 2). Indeed, miR-125a-5p was upregulated only in Treg cells isolated from PLN of patients with T1D, thus confirming the data generated with the microfluidic array cards (Fig. 2A). To understand whether such differential expression was specific for Treg cells residing in the PLN of patients with T1D, miR-125a-5p expression was also tested in T conventional (Tconv) cells purified from PB and from PLN. In contrast to what observed in purified Treg cells, miR-125a-5p was not upregulated in Tconv cells isolated from PLN of patients with T1D (Fig. 2B). Finally, to avoid any potential age-related confounding effects on miR-125a-5p expression levels in PLN Treg cells, a correlation between donors age and miR-125a-5p expression was performed, thus demonstrating that its expression does not depend on age (Supplementary Figure 4).

Collectively, these data demonstrate that hyperexpression of miR-125a-5p is a key feature of Treg-cells isolated from the pancreas draining lymph nodes of patients with T1D. Moreover, this hyperexpression cannot be attributed to a more activated status of Treg cells isolated from PLN of patients with T1D, as we previously showed that these cells do not express higher CD25 levels vs their circulating counterpart or vs Treg in lymph nodes of control subjects^10^.

### MiR-125a-5p targets TNFR2 and CCR2

**Figure 3:**
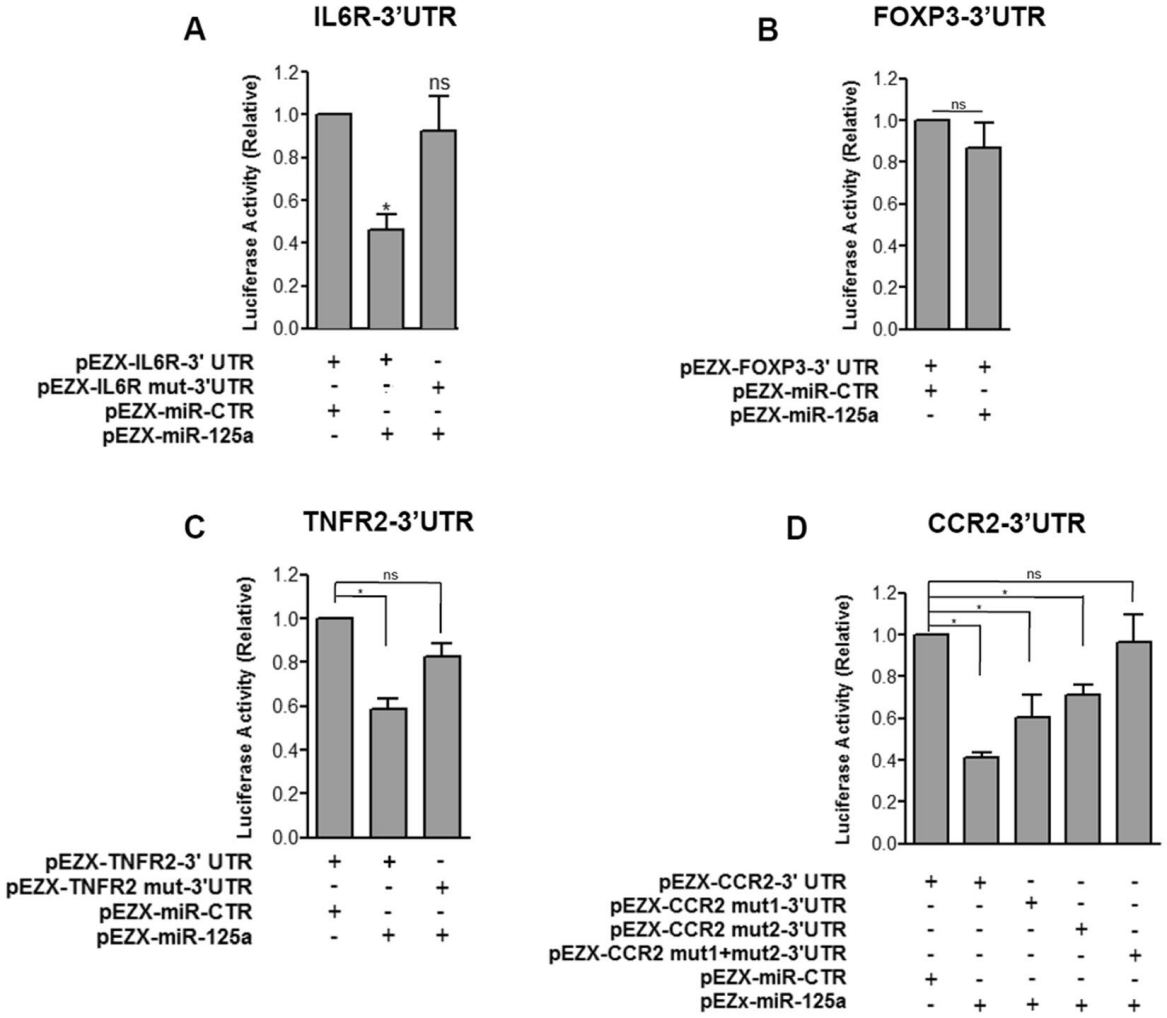
TNFR2 and CCR2 are targets of microRNA miR-125a-5p. Luciferase activity in HeLa cells transfected with (**A**) IL6R (wt, mutated)-, (**B**) FOXP3-, **(C)** TNFR2 (wt, mutated)- or (**D**) CCR2-3′UTR (wt, mutated)-Firefly Luciferase reporter plasmid together with miR-125a-5p or with scrambled microRNA expressing vector. Firefly luciferase activity was divided by the *Renilla* luciferase activity to correct eventual differences in transfection efficiency. Bars represent mean + SD of 3 independent experiments. *p < 0.05 paired student’s *t test*.

MicroRNA miR-125a-5p was previously shown to modulate human Treg cell function^13^. Changes in miR-125a-5p expression levels have been demonstrated to modify sensitivity of Treg cells to cytokines, therefore modulating their phenotype and response. To gain insights into further potential functional alterations that may be caused by miR-125a-5p upregulation in PLN-residing Treg cells of patients with T1D, a computational prediction analysis of miR-125a-5p target genes was performed. Two different algorithms were used: Targetscan 6.2 and PICTAR. Based on the unified results retrieved, a list of target genes specifically belonging to at least one of the following functional categories was generated: T-cell and Treg-specific function, apoptosis, cytokine and chemokine signaling pathways and adhesion or migration functions (Table 1). Using luciferase assay, we confirmed the previously demonstrated binding of miR-125a-5p to IL6R 3′UTR (Fig. 3A). Furthermore, among additional putative miR-125a-5p target genes of potential interest, we selected FOXP3, TNFR2 (Tumor Necrosis Factor Receptor Type II) and CCR2 (C-C Chemokine Receptor type-2) with potential target sites within their 3′UTR sequences (Supplementary Figure 5), to establish whether they indeed were actual target of miR-125a-5p. To this end, luciferase reporter assays were performed. Co-transfection of miR-125a-5p expressing vector and luciferase-FOXP3–3′UTR reporter plasmid in HeLa cells did not result into a reduced luciferase activity, demonstrating that FOXP3 is not a direct target of miR-125a-5p (Fig. 3B). On the other hand, miR-125a-5p bound effectively to TNFR2 3′UTR (Fig. 3C **)** and CCR2 3′UTR (Fig. 3D **)** and this binding was reduced when seed binding sequences on the 3′UTR regions of both TNFR2 and CCR2 were mutated. Interestingly, CCR2 3′UTRs mutated site-1 (113–119 nucleotides of CCR23′UTR, mut1) and/or site-2 (169–176 nucleotides of CCR2 3′UTR, mut2) [sequence ID: NM_001123396.1] differently contributed to the expression of luciferase-CCR2 3′UTR when taken into consideration individually, while the binding was completely abolished when both sites were mutated, thus demonstrating that miR-125a-5p directly targets CCR2-3′UTR in a highly specific manner (Fig. 3D and Supplementary Figure 5D).

**Table 1:** MicroRNA miR-125a-5p target genes of interest.

Target Gene Name	Sequence Accession ID	Predicted or Validated Target Gene
*CCR2*	*NM_001123396*	*Predicted*
BAK1	NM_001188	Validated
NAIF1	NM_197956	Predicted
TNFSF4	NM_003326	Predicted
FOXD2	NM_004474	Predicted
KLF13	NM_015995	Validated
IRF4	NM_001195286	Predicted
MCL1	NM_001197320	Predicted
*IL6R*	*NM_000565*	*Validated*
ETS1	NM_001143820	Predicted
BCL2	NM_000633	Predicted
BCL2L2	NM_001199839	Predicted
*TNFRSF1B (TNFR2)*	*NM_001066*	*Predicted*
KLF3	NM_016531	Predicted
SMAD4	NM_005359	Validated
IL16	NM_004513	Predicted
STAT3	NM_003150	Predicted
CASP2	NM_032982	Predicted
IKZF4	NM_022465	Predicted
*FOXP3*	*NM_001114377*	*Predicted*

Predicted or Validated miR-125a-5p target genes relevant for T-cell and Treg-specific function, apoptosis, cytokine and chemokine signaling pathways, adhesion or migration functions are reported. Official target gene name alongside with Sequence Accession ID are indicated in the table. Target genes taken into consideration in the present study are reported in Italic.

### MiR-125a-5p regulates CCR2 expression in PLN Treg cells from T1D patients

**Figure 4:**
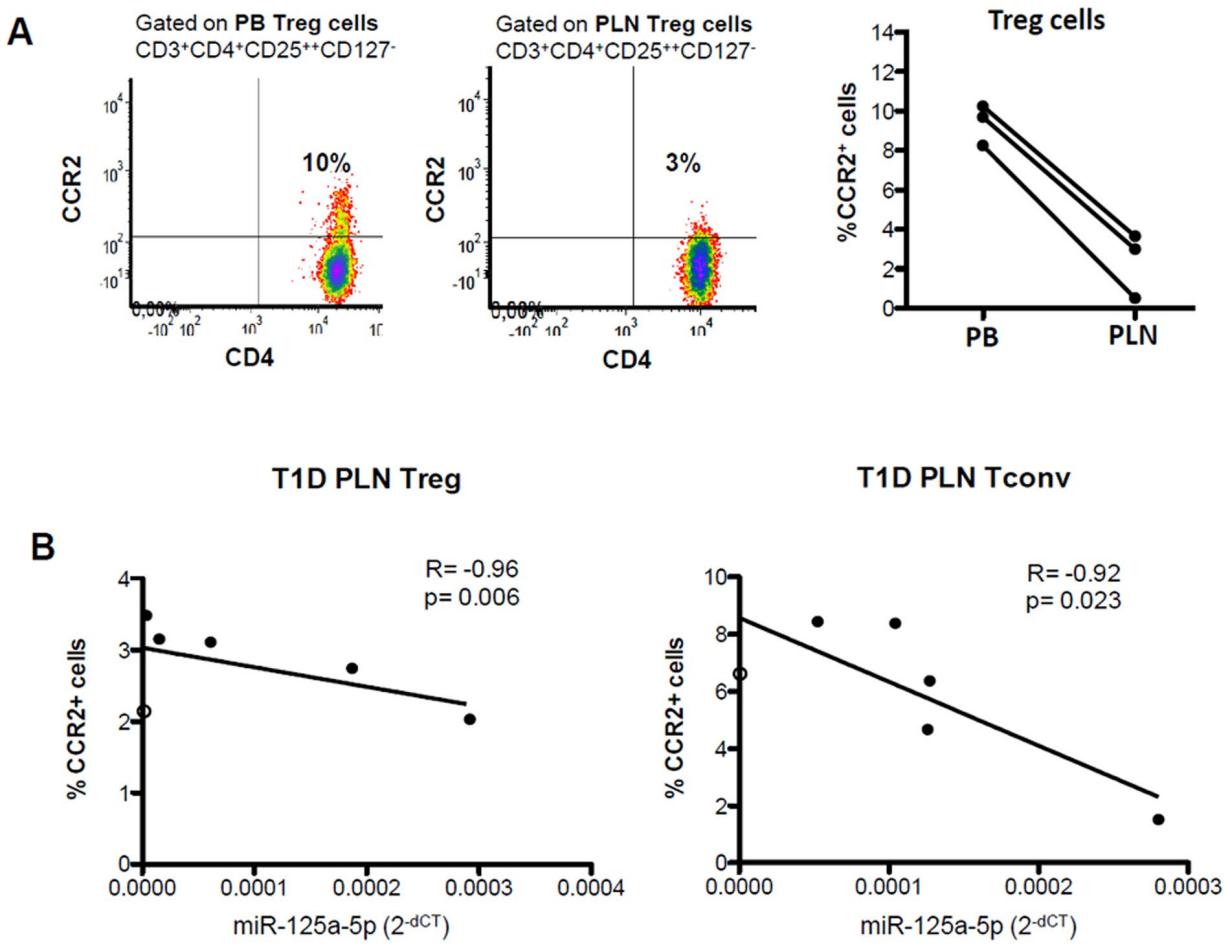
(**A**) Representative flow cytometry dot plot showing CCR2 expression on Treg cells in peripheral blood (PB) and pancreatic lymph nodes (PLN) of a patient with T1D (left panel) and data collected in 3 patients with T1D (right panel). **(B)** Correlation analysis between percentages of CCR2^+^ cells in Treg cells (left panel) and Tconv cells (right panel) measured by flow cytometry and miR-125a-5p expression values measured as 2^−dCT^. Spearman R test was used.

Among analyzed miR-125a-5p target genes, CCR2 was taken into consideration for further experiments: *i*) because CCR2 is the receptor of CCL2 (MCP1) previously suggested to be associated to T1D pathogenesis; *ii)* CCR2 expression in Treg cells modulates their function by modifying their capacity to migrate to inflamed sites and to suppress immune cell activity, thus potentially contributing to impaired Treg cells function in PLN of T1D patients. Therefore, should miR-125a-5p directly modulate CCR2 expression, Treg cells isolated from PLN of patients with T1D would have a reduced CCR2 expression as compared to those isolated from their peripheral blood which could impair their ability to migrate. Flow cytometry data revealed a reverse CCR2 expression on Treg cells isolated from PB and PLN of patients with T1D, as observed for miR-125a-5p expression but in the opposite direction (Fig. 4A). MicroRNA miR-125a-5p expression and frequency of CCR2^+^ cells in Treg and Tconv cells purified from PLN of patients with T1D demonstrated an inverse correlation between miR-125a-5p and CCR2 expression (Fig. 4B). Overall these data show that miR-125a-5p targets CCR2 and its expression is finely tuned in Treg cells.

### The CCR2-ligand CCL2 is specifically enriched in beta-cells in non-diabetic and in T1D patients

**Figure 5:**
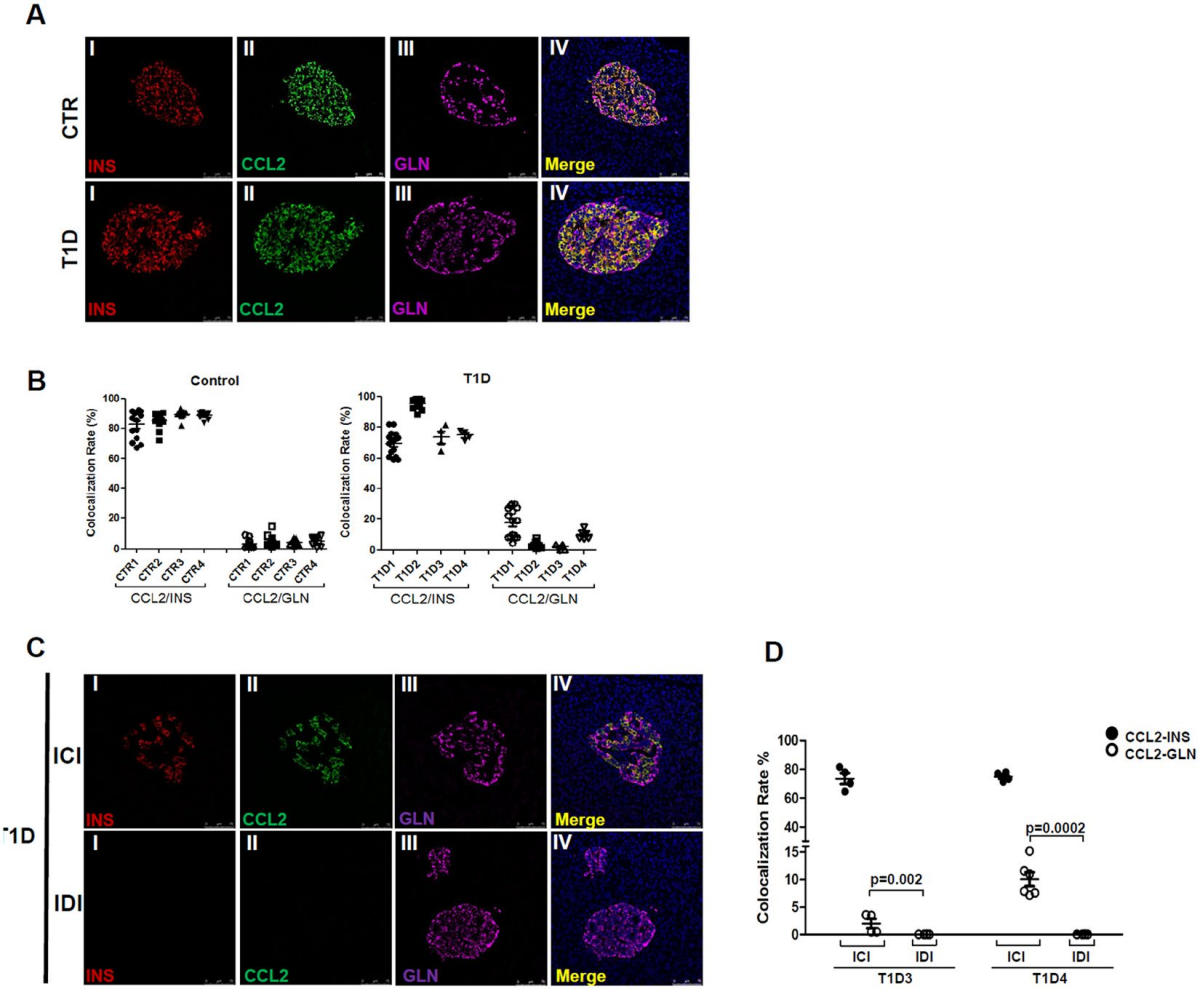
CCL2 is primarily expressed by insulin-producing cells in pancreatic islets. (**A**) Representative images of CCL2 immunofluorescence staining on human pancreatic sections from non-diabetic donors (upper panels) and from subjects with T1D (lower panels). Overlapping signals between CCL2 (green) and insulin (red) staining is shown in panel IV; low/absent overlap was seen with CCL2 and glucagon (magenta) stainings. Scale bar 75 µm. (**B**) Colocalization analysis between CCL2/Insulin and CCL2/Glucagon in non-diabetic donors (left panel) and in patients with T1D (right panels). Values are reported as mean ± SEM of the percentage of colocalization rate values. Each dot represents a single colocalization rate value obtained per single analyzed islet. (**C**) Representative images of CCL2 immunofluorescence on human pancreatic sections from T1D donors in Insulin-Containing Islets (ICI)- upper panel- and in Insulin-Deficient Islets (IDI)- lower panel- Scale bar 75 µm. (**D**) Colocalization analysis between CCL2/Insulin (black filled circle) and CCL2/Glucagon (empty circle) in two T1D donors by separately taking into consideration ICI and IDI. Values are reported as mean ± SEM of the percentage of colocalization rate values. Each dot represents a single colocalization rate value obtained per single analyzed islet. p < 0.05 non-parametric *Mann-Whitney* U *test*.

To our knowledge this is the first report of CCR2 expression evaluation on human Treg cells in PLN of T1D patients and this might be relevant for *in vivo* Treg cell migration in such disease context. Distinct expression of chemokine receptors on T cells is indeed responsible for lymphoid homing versus variable peripheral tissue trafficking. CCL2– i.e. the CCR2 ligand - was then measured on pancreas sections from non-diabetic organ donors and from patients with T1D obtained from the nPOD and the Siena cohorts (donor characteristics in Supplementary Table 3). It is known that pancreatic islets produce and secrete CCL2^14^ but here we provide evidence that CCL2 expression is specifically enriched in insulin-producing cells respect to glucagon-producing cells to a similar extent in non diabetic and in diabetic donors (Fig. 5A and B). Furthermore, the analysis of CCL2 expression pattern in insulin-containing islets (ICI) vs insulin-deficient islets (IDI) in T1D donors showed: *i)* the absence of CCL2 expression in IDI (Fig. 5C), *ii*) the increased expression of CCL2 in glucagon-producing cells within ICI respect to those present in IDI (p < 0.05), indicating that the presence of residual beta-cells triggers inflammation in other islet-endocrine cells also. These new data suggest that, firstly, CCL2 is primarily expressed by pancreatic beta-cells and might serve as potential attractant of CCR2-expressing cells and, secondly, that beta-cells guide the inflammation process which can be broaden to nearest non-beta cells thus amplifying the damage.

## Discussion

Our findings demonstrate, for the first time, a differential expression of miR-125a-5p in Treg cells isolated from pancreas draining lymph nodes of patients with T1D and this might account for the reduced expression of CCR2.

The microRNA expression profiles of human Treg cells have already been reported, but only in Treg cells isolated from the peripheral blood and always analyzed in comparison with Tconv cells. Rouas *et al*. reported a human Treg-cell specific microRNA signature, distinguished from that found in Tconv cells, and miR-125a-5p was significantly under-represented in Treg cells isolated from human cord blood^6^. We observed the same miR-125a-5p distribution in functional Treg and Tconv cells isolated from blood of control non diabetic donors, thus suggesting that miR-125a-5p expression should be low in functional Treg cells and that miR-125a-5p upregulation could be detrimental for Treg-cell function. Accordingly, in our study miR-125a-5p was upregulated only in Treg cells isolated from PLN of patients with T1D and these Treg cells have impaired *in vitro* regulatory functions, as previously shown^3^ and confirmed in this study.

In line with our findings, Treg cells isolated from blood of patients with asthma, a disease characterized by the break of peripheral tolerance and Treg-cell impairment, express high levels of miR-125a-5p^13^. Interestingly, miR-125a-5p is also dysregulated in peripheral CD4+ T cells from patients with systemic lupus erythematous and Crohn’s disease^15, 16^. Here, we show for the first time that miR-125a-5p is dysregulated also in another autoimmune disease, T1D. Specifically, miR-125a-5p is upregulated in Treg cells deriving from the PLN of diabetic patients and not in Treg cells deriving from their peripheral blood counterpart, thus underlining the importance of assessing Treg cells studies in the context of the organ targeted by the autoimmune process rather than the circulation. Such results were obtained in PLN from a first exploratory cohort of 3 T1D patients and confirmed by single assay RT-PCR in a second independent cohort of 6 T1D donors.

It is known that miR-125a-5p is a key factor for Treg-cell function through the regulation of IL-6R and STAT3 expression^13^ and also of other pro-inflammatory genes^16^. Several studies previously reported the effects of miR-125a-5p on Treg cell phenotype; indeed, it has been demonstrated that alteration of miR-125a-5p expression levels lead to impaired sensitivity of Treg cells to cytokines and other stimuli thus rendering miR-125a-5p a node for Treg cell function during immune responses. As a matter of fact, among miR-125-5p target genes, three main cytokine/chemokine receptors were identified: IL6R, TNFR2 and CCR2. We confirmed IL6R as a target of miR-125a-5p, but we also report, for the first time to our knowledge, that TNFR2 and CCR2 are regulated by miR-125a-5p. In particular, CCR2 has two evolutionary conserved binding sites for miR-125a-5p in its 3′UTR and they differently contribute to CCR2 luciferase expression. In addition, concomitantly with miR-125a-5p upregulation, the percentage of CCR2+ Treg cells purified from PLN of T1D patients was decreased, thus showing how miR-125a-5p fine-tunes CCR2 expression in Treg cells.

CCR2 was shown to be expressed also in murine Treg cells^17, 18^ and this expression is crucial for homing mechanisms induced by specific chemokines secreted by the inflamed tissues^19, 20^. As a matter of fact, it has been demonstrated that Treg cells are active in insulitic lesions of NOD mice pancreas and are not just restricted in draining lymph nodes^21^; moreover, their activity at the inflammatory site is essential in order to suppress autoimmune responses. Whilst many studies have investigated the role of Treg cells in insulitic lesions of NOD mice, reports in human pancreas are limited in numbers. One study examined the presence of FOXP3+ Treg cells in post-mortem pancreas samples from patients with recent-onset T1D, despite the abundance of CD4+ and CD8+ T cells, FOXP3+ Tregs were barely detected in islets suggesting the lack/reduction of regulatory cells at the site of autoimmune attack^22^. However, Treg cells can potentially migrate to the pancreas as reported in pancreatic cancer studies^23, 24^. Indeed, human antigen-primed Treg cells upregulate the expression of chemokine receptors once residing in secondary lymphoid tissues in order to acquire the ability to migrate to non-lymphoid target organs under the stimuli of chemoattranct stimuli and following CCL2 gradients^25, 26^. In support of this, our data demonstrate the expression of CCL2 (MCP-1) -the CCR2-ligand- primarily in pancreatic beta-cells of T1D organ donors, suggesting the beta-cell release of chemoattractant of CCR2-expressing cells.

Although, miR-125a-5p has been reported to be a central node for Treg cell function, its hyper-expression in Treg cells derived from T1D PLN could be positioned in a wider context of immune cell dysfunction, possibly contributing to the lack of immune-tolerance in T1D. Therefore, microRNA miR-125a-5p upregulation could represent one of those disrupted mechanisms which are highly required by Treg cells to fully exert their immune surveillance role and, together with other altered factors, contributing to their dysfunction. Indeed, other non-coding RNAs not analyzed in the present study might be relevant for Treg cell dysfunction in T1D context and future studies are needed to fully elucidate the complete non-coding RNAs expression fingerprint in Treg cells derived from T1D PLN.

In conclusion, the aberrant up-regulation of miR-125a-5p in Treg cells of patients with T1D when residing in the pancreatic draining lymph nodes, possibly due to local inflammation or disease-specific signals, leads to reduced CCR2 expression and this may impede the pancreas-specific migration of Treg cells, thus depriving this organ of immune cells key for maintenance of peripheral tolerance.

## Methods

### Donor and sample collections

This study was approved by the San Raffaele Hospital Ethics Committee (protocol DRI-003). Blood and pancreatic lymph nodes were collected as previously described^10^. Donor characteristics are reported in Supplementary Tables 1 and 2. Cells were isolated as previously described^10^. nPOD (Network for Pancreatic Organ Donors) non-diabetic control donors (case ID: 6098, 6153, 6024, 6174) and T1D donors pancreatic sections (case ID: 6052, 6113) and 2 T1D donors pancreata sections from Siena cohort, were included in the analysis (Supplementary Table 3).

### MicroRNA expression profiles and target-gene prediction

Two-hundred Treg and Tconv cells were purified by fluorescence-activated cell sorting (Supplementary Figure 1) and microRNAs were analyzed with the human Megaplex RT-stem-loop microRNA Pool A v2.1 (Life Technologies, CA-USA). Expression of hsa-miR-125a-5p (here named miR-125a-5p) was also tested by TaqMan microRNA single assay qPCR on preamplified products using the following assays: hsa-miR-125a-5p (ID002198), snRNAU6 (ID001973), snRNAU44 (ID001094), and snRNAU48 (ID1006) (Life Technologies). Expression levels of each microRNA are reported as Cycles to Threshold (Ct) of PCR and Ct are normalized (dCt) using small RNAs endogenous controls (RNU6A, RNU48, RNU44). MicroRNAs were considered differentially expressed when the cutoff fold change was <0.5 or >2.0 and when the cutoff p-value was <0.05 using the 2-tailed “Student t-test” on normally distributed dCt values. Undetermined values were set to a maximum Ct of 40. Each amplification plot for every microRNA was manually checked to avoid false positive Ct and only microRNAs with a Ct <35 and with adequate efficiency amplification plots in all sample replicates were taken into consideration for 2^−dCT^ calculations and subsequent statistical analyses. Targetscan6.2 and PICTAR algorithms were used to identify potential microRNA target genes.

### Luciferase assay

HeLa cells were transfected with dual luciferase reporter plasmids, either pEZX-MT01-CCR2-3′UTR (HmiT054686-MT01), pEZXMT01-FOXP3-3′UTR (HmiT012269-MT01), pEZXMT01-TNFR2-3′UTR (HmiT018149-MT01), pEZXMT01-IL6R (HmiT009672-MT01) together with a precursor expressing vector pEZX-MR04-miR-125a-5pplasmid (HmiR0309-MR04) (Genecopoeia, Rockville, USA). pEZX-MR04-miR scramble vector (cmiR-0001-MR04) was used as control.

125 ng of total DNA was transfected at a ratio of 1:50 (3′UTR:miRNA). The miR-125a binding site in the 3′UTR of validated target genes were mutated by using Quick Change mutagenesis kit (Agilent technologies, Santa Clara, CA, USA) according to manufacturer instructions. HeLa cells were harvested 48 h post-transfection and renilla luciferase activities were measured using Dual Luciferase Reporter assay (Promega, Fitchburg, WI, USA) GLOMAX 20/20 luminometer (Promega).

### Functional studies and phenotype analysis

The suppressive function of purified Treg cells was measured as previously described^10^. The phenotype analysis was performed by flow cytometry by staining the cells with anti-CD3, -CD4, -CD25, -CD127 mAbs and 7AAD for cell sorting and with anti-CD3, -CD4, -CD25, -CD127, and -CCR2 for surface stainings. All monoclonal antibodies were obtained from Beckman Coulter (Brea, CA-USA) except for the anti-CCR2 (R&D Systems, Minneapolis, MN-USA). Samples were analyzed using the BD FACS Canto II (Becton Dickinson, San Jose, CA)and sorted using the MoFlo XDP (Beckman Coulter, USA).

### Immunofluorescence analysis

Triple immunofluorescence staining of formalin-fixed and paraffin-embedded pancreatic specimens was performed using polyclonal rabbit anti-CCL2 (Abcam, Cambridge Sciences Park, Cambridge, UK), polyclonal guinea pig anti-insulin (Dako, Glostrup, Denmark), and monoclonal mouse anti-glucagon (R&D Systems, Minneapolis, MN) and secondary Alexa-Fluor labeled antibodies (Life Technologies). Donor characteristics are depicted in Supplementary Table 3. After nuclear staining with DAPI, slides were analyzed with a Leica TCS SP5 laser scanning confocal microscope and colocalization analysis was performed with Leica LAS-AF software (Leica Microsystems GmBH, Wetzlar, Germany).

### Statistical analysis

Comparisons between groups were performed using the two-tailed Student t test or the Mann-Whitney U test for non-parametric data. For the analysis of paired samples in the suppression assay, the Wilcoxon test was applied. Linear regression was used to determine the correlation between variables. For all analyses, a two-tailed P value < 0.05 was considered significant. Statistical analyses were performed using Stata 10.1 software (Stata Corp., College Station, TX).

### Ethical Approval and Informed Consent

All experimental protocols were approved by the San Raffaele Hospital Ethics Committee (protocol DRI-003). All procedures followed were in accordance with the ethical standards of the responsible committee on human experimentation (institutional and national) and with the Helsinki Declaration of 1975, as revised in 2008. Informed consent was obtained from all patients for being included in the study.

## Electronic supplementary material

Supplementary info
