# Supplementary material for: Regulatory T-cells from pancreatic lymphnodes of patients with type-1 diabetes express increased levels of microRNA miR-125a-5p that limits CCR2 expression
Source: Sci Rep. 2017 Jul 31;7:6897. doi: 10.1038/s41598-017-07172-1 (PMC5537269; doi:10.1038/s41598-017-07172-1)
Supplement: Supplementary file 1 — Supplementary info [file 41598_2017_7172_MOESM1_ESM.doc]

**SUPPLEMENTARY INFO**

**Regulatory T-cells from pancreatic lymphnodes of patients with type-1 diabetes express increased levels of microRNA miR-125a-5p that limits CCR2 expression**

Guido Sebastiani^1,2*^, Giuliana Ventriglia^1,2*^, Angela Stabilini^3^, Carlo Socci^4^, Cristina Morsiani^3,‡^, Andrea Laurenzi^3,5^, Laura Nigi^1,2^, Caterina Formichi^1,2^, Bechara Mfarrej^3^, Alessandra Petrelli^3^, Georgia Fousteri^3^, Todd M. Brusko^6^, Francesco Dotta^1,2§^ and Manuela Battaglia^3§^

^1^Diabetes Unit, Department of Medicine, Surgery and Neuroscience, University of Siena. Siena, Italy

^2^Fondazione Umberto Di Mario ONLUS c/o Toscana Life Science, Siena, Italy.
^3^Diabetes Research Institute, IRCCS San Raffaele Scientific Institute, Milan, Italy.
^4^Department of Surgery, IRCCS San Raffaele Hospital, Milan, Italy.
^5^Department of Internal Medicine, IRCCS San Raffaele Hospital, Milan, Italy.

^6^Department of Pathology and Laboratory Medicine, Diabetes Institute, Gainesville, FL, USA.

^‡^current address: Interdepartmental Centre "L. Galvani" for Integrated Studies of Bioinformatics, Biophysics and Biocomplexity (CIG), University of Bologna , Bologna.

^*^These authors contributed equally to this work.

^§^These authors share senior Authorship

*Correspondence to:*

Prof. Francesco Dotta

Diabetes Unit, Dept. of Medicine, Surgery and Neurosciences,

University of Siena, Siena, Italy

Tel. +39-0577-586269

FAX: +39-0577-586186

Email: francesco.dotta@alice.it

**Supplementary Figure legends**

**Figure 1S. (A) Sorting strategy and purity of Treg and Tconv cells.** Cells were first gated for singlets (FSC-H vs. FSC-A) and lymphocytes (SSC-A vs. FSC-A). The lymphocyte gate was further analyzed for cell uptake of the Live/Dead 77AD stain and CD4 expression to take only the live, healthy CD4+ T cells (7AAD^-^, CD4^+^). CD25 and CD127 surface expression was then determined from this gated population to purify Treg cells (CD25^++^CD127^-^) and Tconv cells (CD25^-^CD127^+^). (**B**) microRNA profiles in 200 and in 5000 Treg cells**.** Mean microRNA expression values in Treg cells purified from 3 independent control non-diabetic donors measured as 2^-dCT^. The red dot/arrow highlight miR-125a-5p expression values. R-value and p-value were calculated using Spearman R test.

**Figure 2S. MicroRNA miR-31 and miR-125a-5p analysis in PB Tconv and PB Treg.** Real Time PCR analysis of 200 sorted Tconv and Treg cells from PB of 3 healthy controls (HC) non-diabetic subjects. Expression values are reported as mean+SD of 2^-delta cycle threshold (2^-dCT^) values normalized using three different small RNAs (RNU6, RNU44, RNU48).

**Figure 3S. Impaired in vitro suppressive capacity of Treg cells isolated from PLN of T1D patients.** Tconv cells were purified as described in Figure 1S and cultured with autologous Treg cells (3:1 ratio) purified from peripheral blood (PB) of 3 non diabetic controls (CTR), pancreatic lymph nodes (PLN) of 3 non diabetic CTR, and from PB and PLN of 3 patients with T1D. The suppressive capacity of purified Treg cells was measured as the ability to inhibit proliferation of Tconv cells cultured in the absence of Treg cells (given an arbitrary value of 100%).

**Figure 4S**. **miR-125a-5p expression in PLN Treg is not correlated to donors age.** MicroRNA miR-125a-5p expression values in Treg cells sorted from non diabetic (n=6) and T1D PLN (n=10) were correlated to donors age (years). Expression values are reported as 2^-delta cycle threshold (2^-dCT^) values normalized using three different small RNAs (RNU6, RNU44, RNU48). Spearman R test was used.

**Figure 5S. miR-125a-5p target genes binding sites.** MicroRNAs miR-125a-5p predicted binding sites within 3’UTR of (**A**) IL6R, (**B**) FOXP3, (**C**) TNFR2 and (**D**) CCR2 mRNA. Binding sites nucleotide position were retrieved from Targetscan6.2.

**Supplementary Figure 1**


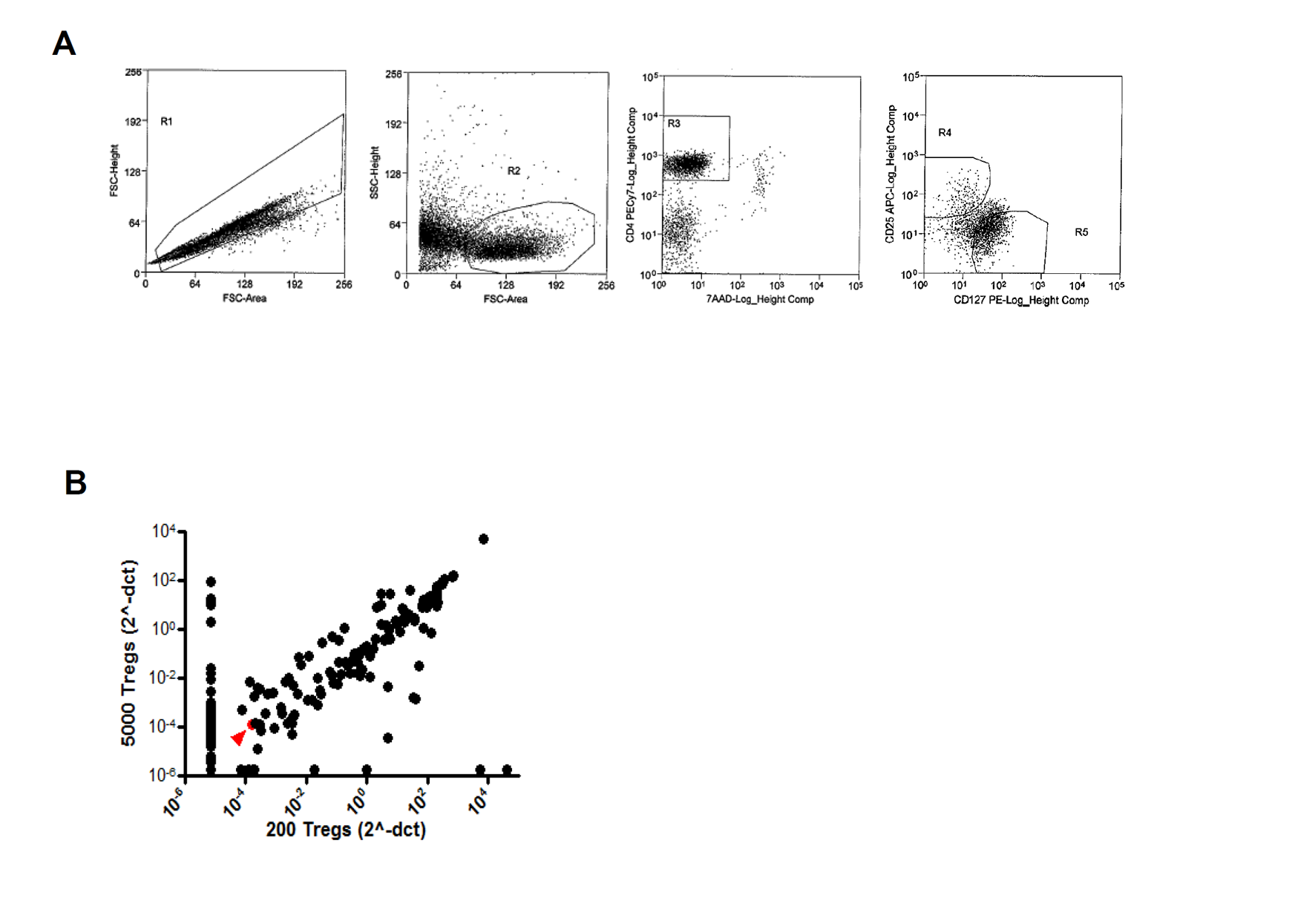


**Supplementary Figure 2**


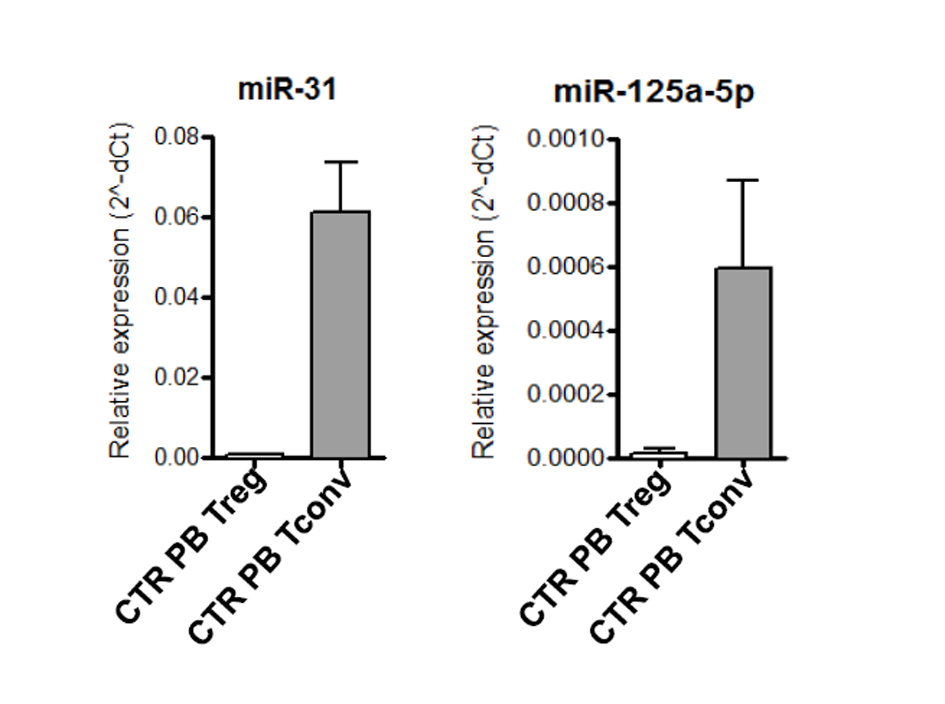


**Supplementary Figure 3**


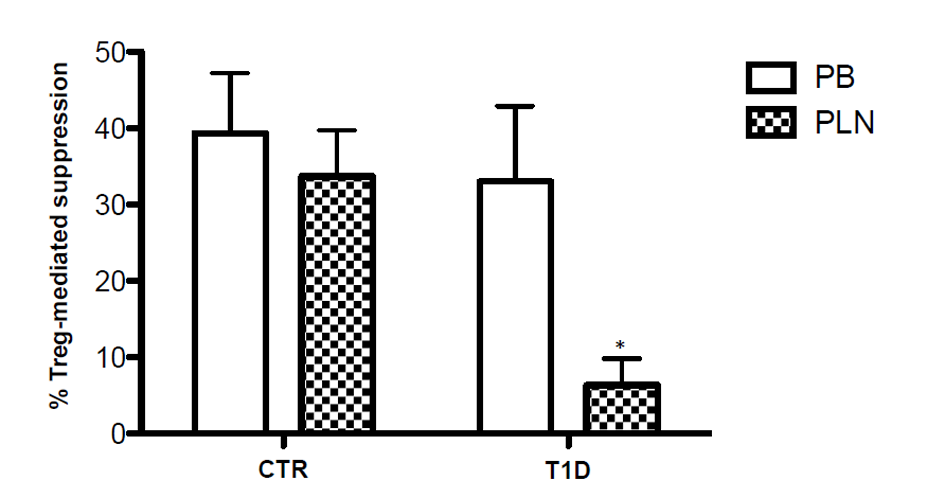


**Supplementary Figure 4**


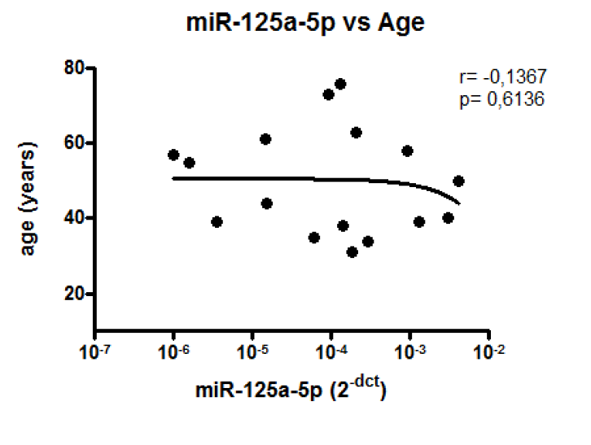


**Supplementary Figure 5**


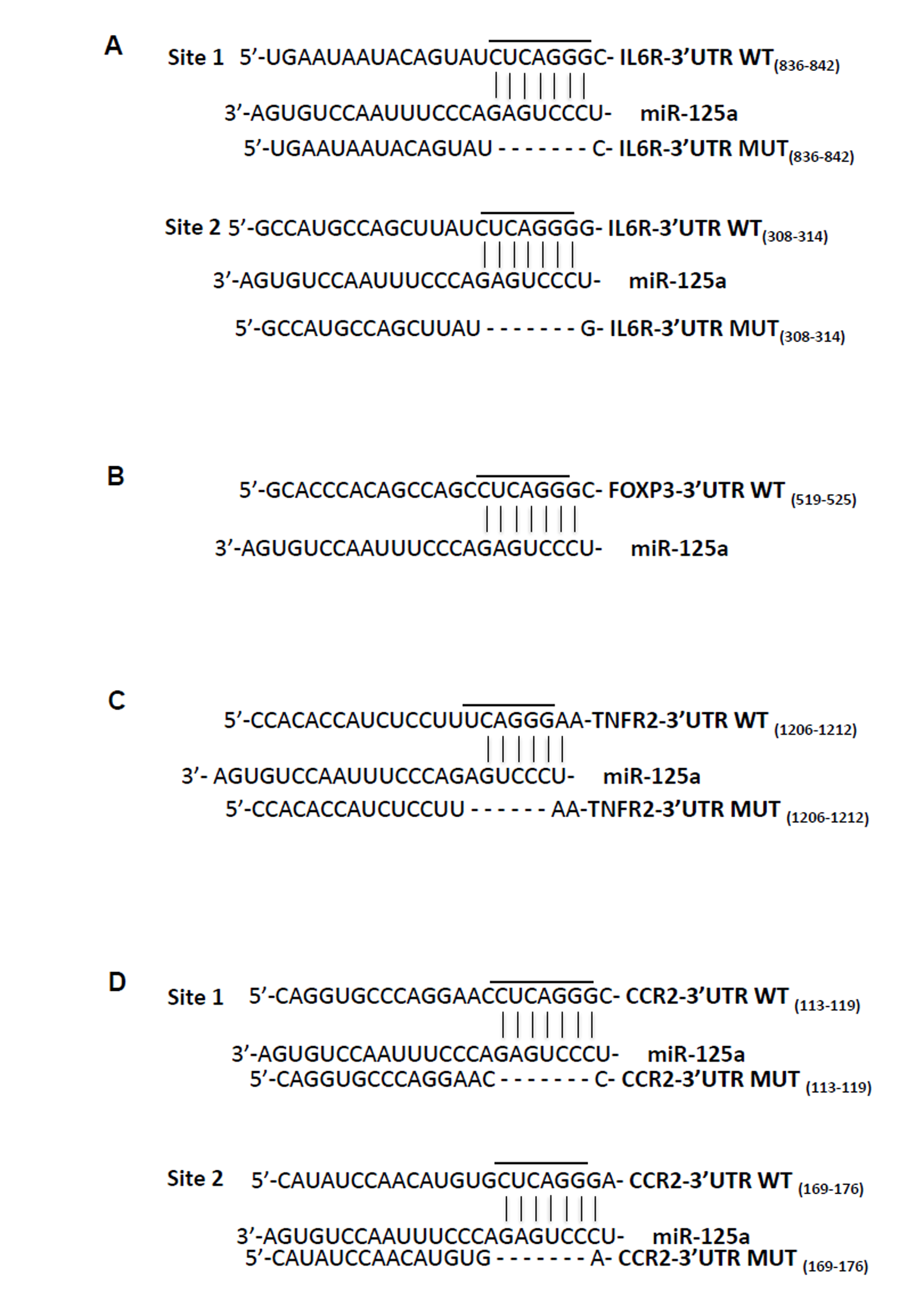


**Supplementary Tables**

**Supplementary Table 1.** *Characteristics of the subjects who donated samples analyzed in Figures 1A-B-C*

|  | **non-diabetic controls** | **non-diabetic controls** | **T1D** |
| --- | --- | --- | --- |
| Sample | Peripheral Blood | Pancreatic  Lymph nodes | Peripheral Blood  Pancreatic Lymph nodes |
| N | 8 | 3 | 4 |
| Age (years) | 36 (28-43) | 66 (58-76) | 42 (39-50) |
| Female sex, n (%) | 4 (50) | 2 (75) | 4 (100) |
| T1D duration (years) | n.a. | n.a. | 20(15-26) |
| HbA1c (%)  (mmol/mol) | n.a. | n.a. | 9.7(8.6-11.6)  83 (71 – 103) |
| T1D-AutoAb pos (%) | n.a. | n.a. | 2 (50%) |

**Supplementary Table 2.** *Characteristics of the subjects who donated samples analyzed in Figures 2A-B*

|  | **non-diabetic controls** | **non-diabetic controls** | **T1D** | **T1D** |
| --- | --- | --- | --- | --- |
| Sample | Peripheral  Blood | Pancreatic Lymph nodes | Peripheral  Blood | Pancreatic Lymph nodes |
| N | 8 | 3 | 8 | 6 |
| Age (years) | 30(26-45) | 64(61-73) | 38 (25-59) | 40 (31-55) |
| Female sex, n (%) | 6 (75) | 1 (25) | 4 (50) | 2 (33) |
| T1D duration (years) | n.a. | n.a. | 19 (7-29) | 27 (22-43) |
| HbA1c (%)  (mmol/mol) | n.a. | n.a. | 9.4(7.8-11)  79 (62 – 97) | 9.6(8-10.9)  81 (64 – 96) |
| T1D-AutoAb pos (%) | n.a. | 2 (50%) | 5 (63) | 4 (67%) |

**Supplementary Table 3.*Characteristics of the subjects who donated samples analyzed in Figures 5A-D***

|  | **non-diabetic controls** | **T1D** |
| --- | --- | --- |
| N | 4 | 4 |
| Age (years) | 19(15-21) | 43 (12-73) |
| Female sex, n (%) | 0 (0) | 2 (50) |
| T1D duration (years) | n.a. | 11 (1-30) |
